# Topics of the Month

**Published:** 1893-12

**Authors:** 


### TOPICS OF THE MONTH.

Consumption was officially declared a communicable disease by the Michigan State Board of Health, at its meeting, held September 30, 1893, and it was decided that it must be reported by physicians and householders to the several boards of health. This we regard as a very timely order, and we hope that it will be technically and efficiently enforced.

SOUTHERN PINES, Moore county, North Carolina, is a new Winter health resort, just coming into prominent notice. It is located in the high, dry, long-leaf pine sand hills, amid the tar, pitch, and turpentine district. Thousands of Northern invalids have visited this region, and many remarkable cures have been effected. Prominent physicians have visited the place for investigation, and, without a single exception, say it is the best in the United States, and we are specially requested by Mr. John T. Patrick, Commissioner of Immigration for the Southern States, to invite physicians of the Northern and Western States to visit the locality and investigate in the interest of their patients. Any physician desiring information can address Mr. Patrick, at Pine Bluff, N. C.

---

AT THE Emergency Hospital at the World's Fair, there were treated 18,500 cases, and there were twenty-three deaths and nine births at the institution. This seems to make a creditable showing for the hospital, where so many people, from all sections of the world, were cared for.

---

AT THE annual meeting of the American Orthopedic Association, recently held in St. Louis, Dr. L. A. Weigel, of Rochester, exhibited a hip splint, with a traction mechanism, which consists of a coarse screw which telescopes into the shaft of the splint, this shaft being constructed of steel tubing. For the purpose of preventing the screw from rotating within this tubing, a feather is braced in, which works in a groove cut in the screw. Traction is obtained by running the screw out by turning a milled nut. When a desired amount of traction is obtained, this nut is firmly held in position by a lock-nut, which prevents all possibility of the screw going back from its position by concussion in walking or other means.

---

THE *Medical News* commented editorially, in its issue of November 18th, on society transactions and libraries in a vein that we most heartily endorse. The editor has had occasion recently to look up a certain subject, on which he hoped to obtain information from the transactions of one of the medical societies of America. He was, however, surprised to learn that the volume desired could not be found in the library of the College of Physicians and Surgeons in Philadelphia. But he also found that the transactions of most of the American societies were conspicuous by their absence from the famous library in question. He then says that every society should instruct its secretary or its editor to send the annual volume to all the medical libraries, and even to some selected general libraries, such as those of historical societies of the respective States and counties. We endorse this suggestion most heartily, and hope that the secretary of the Medical Society of the State of New York will take hint from Dr. Gould's suggestion, and place the annual volume of Transactions in all the great medical libraries of the land.

---

AT LAST the patient and long-suffering citizens of Buffalo who patronize the street cars, are promised relief from the distressing cold of Winter weather while in transit. The street car company, through its manager, Mr. Littell, has given assurance, in a published interview, that stoves have actually arrived in Buffalo, and will be placed in the cars as rapidly as possible. Let us hope that this promise will be kept, and that in future it will be one of the comforts of street railway travel to ride in a warm car. Opposition to this sanitary measure has kept the people from enjoying its benefits and privileges for many years,—an opposition born in ignorance and bred in obstinacy. The JOURNAL has for many years contended for this reform, in the publishing of occasional editorial articles advocating it, and it will be a source of gratification to its management to witness a consummation that is so devoutly to be wished.

---

DR. ROBERT BATTEY, of Rome, Ga., has presented his medical library to the State, and it has been accepted. This will form the nucleus of what will ultimately grow to be a valuable collection of medical works. The example of Dr. Battey is to be highly commended. In our own city, too, Dr. F. W. Bartlett has presented his valuable medical library, consisting of several hundred volumes, to the Buffalo Academy of Medicine. Such generous actions on the part of public-spirited citizens ought not to pass without comment, and they deserve the approbation of all progressive men. We hope that Dr. Bartlett's munificent gift will be supplemented by others, so as to create in Buffalo, within a reasonable time, one of the best medical libraries in the State. It certainly ought to be made second only to that of the New York Academy of Medicine.

The greed of Mr. Anthony Comstock, special agent of the Post-office Department, has at last led him to visit Buffalo and fasten his clutches upon the Daggett Table Company with more zeal than judgment. It is claimed by this apostle of purism that the catalogue which the Daggett Table Company sent out is not in strict accordance with Mr. Comstock's ideas of propriety. The manager of the company was placed under arrest and cited before United States Commissioner Fairchild, when the special agent was allowed to tell his version of the story which led to the arrest.

Unless we are misinformed, this virtuous agent did not confine himself strictly to the rules of propriety in relation to his correspondence with the company. We are also informed that, among other things, he claimed that the company sent out circulars that were unmailable and in violation of the statutes, no matter for what purpose they were intended or used.

Whatever may be the motive behind this venomous attack upon a legitimate business, in this particular instance it is important that the entire medical profession shall understand that, if it succeeds, the whole illustrated literature of medicine may be excluded from the mails. We cannot believe that any court will listen to the puerile sentimentality of this attenuated and hypervirtuous fossil; hence, the most that can come to the Daggett Table Company will be the annoyance that grows out of such citation.

We are in accord with the *Buffalo Express* when it asserts that "Anthony Comstock is engaged in more useful work in prosecuting saloon-keepers who circulate indecent business cards than when he prosecutes physicians for spreading needed medical information."

---

Dr. William B. Dewees, of Salina, Kansas, read a paper, at the late meeting of the Mississippi Valley Medical Association, entitled The Erect Posture for Gynecological Examinations. He said, among other things, that digital examination, per vaginam, with the patient in the erect posture, affords one of the most positive means for diagnosis in gynecology. It is a well-established fact that respiration, the various movements and attitudes of the body, as well as pathological conditions, change the conditions and environments of the viscera. Thus the importance of posturing the patient in making physical examinations in gynecic practice becomes evident, as most of the symptoms of diseases of the intrapelvic organs are more marked, and very many only manifested when the patient is standing; while certain conditions of descent, prolapse, or displacement may entirely disappear or change when the pressure or the superincumbent weight of the abdominal viscera is removed by the patient being placed in the dorsal, semi-prone, genu-pectoral, or high pelvic positions; therefore, the erect posture is of paramount importance as an aid in diagnosis in this field of labor.

The author emphasized the advantage and necessity of digital examination in the erect posture, more particularly in examinations undertaken for a cure in women of—first, displacements of the uterus; second, vesical and rectal disorders; third, lack of perineal and vaginal support; fourth, ovarian and tubal disorders; fifth, abdominal and pelvic tumors; and sixth, differentiation between abdominal tumors and pregnancy.

---

THE Orleans Parish Medical Society, following the lead of a few of the foremost medical organizations of the land, has adopted the plan of sending galley proofs of its proceedings to the medical journals. We desire to commend this progressive spirit, and wish that some of the societies near home might follow the example of this spirited Louisianan. If more pains were taken to publish full reports of discussions, in connection with papers previously put in type and distributed in galleys to the members, it would increase the value of society work four-fold.

---

THE *Medical Mirror*, in its November issue, has damned Mr. Ernest Hart with faint praise, but "let us judge not that we be not judged." The *Post-Graduate*, for November, also pays its respects to Mr. Hart in its usual forceful, logical, and convincing manner. Both these journals ought to be read on the subject to be appreciated. We tender, in this connection, our thanks to the erudite and brainy editor of the *Ohio Medical Journal* for its complimentary reference to us in its November number. *Vale*, Mr. Ernest Hart!

